# Supplementary material for: Trabectedin and Lurbinectedin Extend Survival of Mice Bearing C26 Colon Adenocarcinoma, without Affecting Tumor Growth or Cachexia
Source: Cancers (Basel). 2020 Aug 17;12(8):2312. doi: 10.3390/cancers12082312 (PMC7463843; doi:10.3390/cancers12082312)
Supplement: Supplementary file 1 [file cancers-12-02312-s001.pdf]

*cancers*

MDPI

*Article*

# Trabectedin and Lurbinectedin Extend Survival of Mice Bearing C26 Colon Adenocarcinoma, without Affecting Tumor Growth or Cachexia

**Giorgio Aquila [1,†], Andrea David Re Cecconi [1,†], Mara Forti [1], Roberta Frapolli [2], Ezia Bello [2], Deborah Novelli [3], Ilaria Russo [3], Simonetta Andrea Licandro [2], Lidia Staszewsky [3], Giulia Benedetta Martinelli [1], Laura Talamini [4], Laura Pasetto [4], Andrea Resovi [5], Raffaella Giavazzi [2], Eugenio Scanziani [6,7], Giorgia Careccia [8], Emilie Vénéreau [8], Serge Masson [3], Roberto Latini [3], Maurizio D'Incalci [2] and Rosanna Piccirillo [1,*]**

1 Department of Neurosciences, Mario Negri Institute for Pharmacological Research IRCCS, 20156 Milan Italy; giorgio.aquila@marionegri.it (G.A.); andrea.rececconi@marionegri.it (A.D.R.C.); forti9410@gmail.com (M.F.); giulibenny@hotmail.it (G.B.M.); rosanna.piccirillo@marionegri.it (R.P.)

2 Department of Oncology, Istituto di Ricerche Farmacologiche Mario Negri IRCCS, 20156 Milan, Italy; roberta.frapolli@marionegri.it (R.F.); ezia.bello@marionegri.it (E.B.); s.licandro@italfarmaco.com (S.A.L.); raffaella.giavazzi@marionegri.it (R.G.); maurizio.dincalci@marionegri.it (M.D.)

3 Department of Cardiovascular Medicine, Istituto di Ricerche Farmacologiche Mario Negri IRCCS, 20156 Milan, Italy; deborah.novelli@marionegri.it (D.N.); russilar9@gmail.com (I.R.); lidia.staszewsky@marionegri.it (L.S.); serge.masson76@gmail.com (S.M.); roberto.latini@marionegri.it (R.L.)

4 Department of Biochemistry and Molecular Pharmacology, Istituto di Ricerche Farmacologiche Mario Negri IRCCS, 20156 Milan, Italy; laura.talamini@marionegri.it (L.T.); laura.pasetto@marionegri.it (L.P.)

5 Department of Oncology, Istituto di Ricerche Farmacologiche Mario Negri IRCCS, 24126 Bergamo, Italy; andrea.resovi@marionegri.it (A.R.)

6 Dipartimento di Medicina Veterinaria, Università di Milano, 20133 Milan, Italy; eugenio.scanziani@unimi.it (E.S.)

7 Mouse and Animal Pathology Lab (MAPLab), Fondazione UniMi, Università di Milano, 20139 Milan, Italy;

8 Division of Genetics and Cell Biology, IRCCS San Raffaele Scientific Institute, 20132 Milan, Italy; careccia.giorgia@hsr.it (G.C.); venereau.emilie@hsr.it (E.V.)

* Correspondence: rosanna.piccirillo@marionegri.it; Tel.: +39-02-39014371

† These authors contributed equally to this work.

## Supplementary Figures

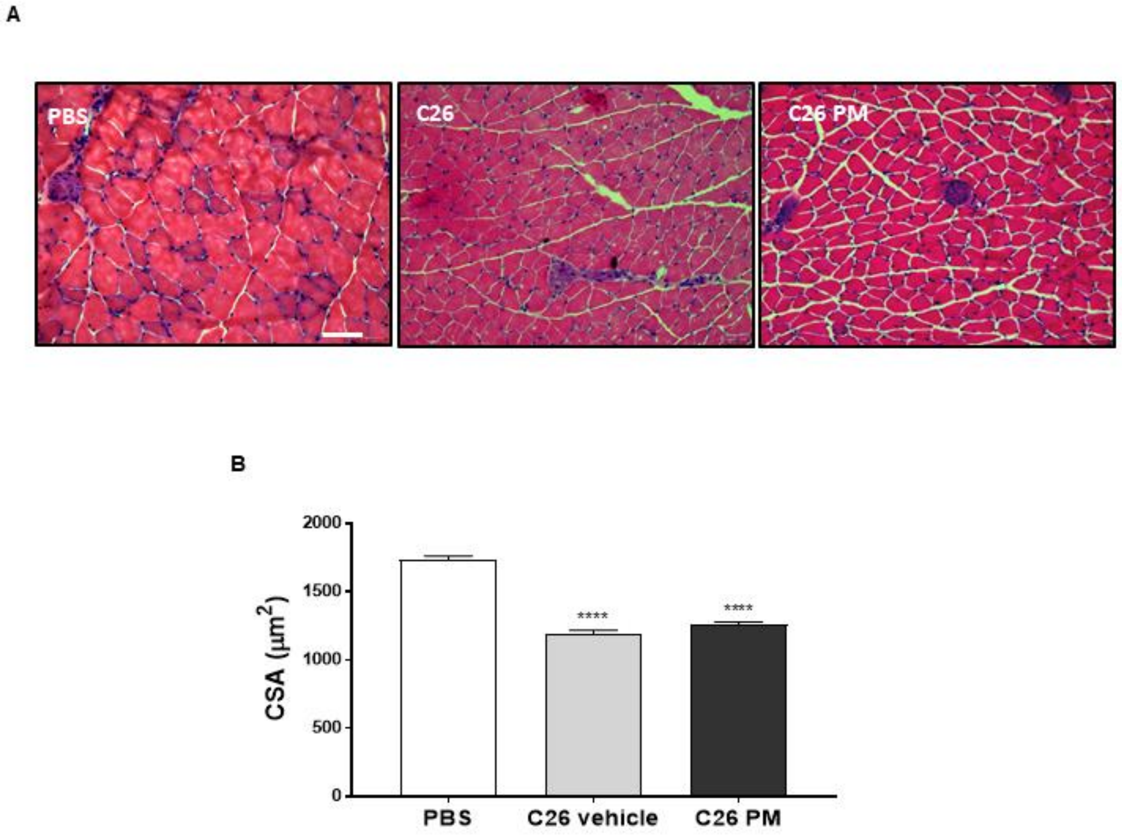


**Figure S1. PM01183 does not prevent fiber atrophy of the gastrocnemii.** Representative Hematoxylin and Eosin-stained images of transversal sections of gastrocnemii from mice injected with PBS, C26 vehicle or C26 PM01183 (C26 PM) are shown (scale bar: 50μm) (A). The mean cross-sectional area (CSA) of fibers of 3-4 muscles per group was measured manually with ImageJ software (B). All results are plotted as mean ± SEM, n = 287-994. **** $p \leq 0.0001$, Kruskal-Wallis with post hoc Dunn's multiple comparison test (B).

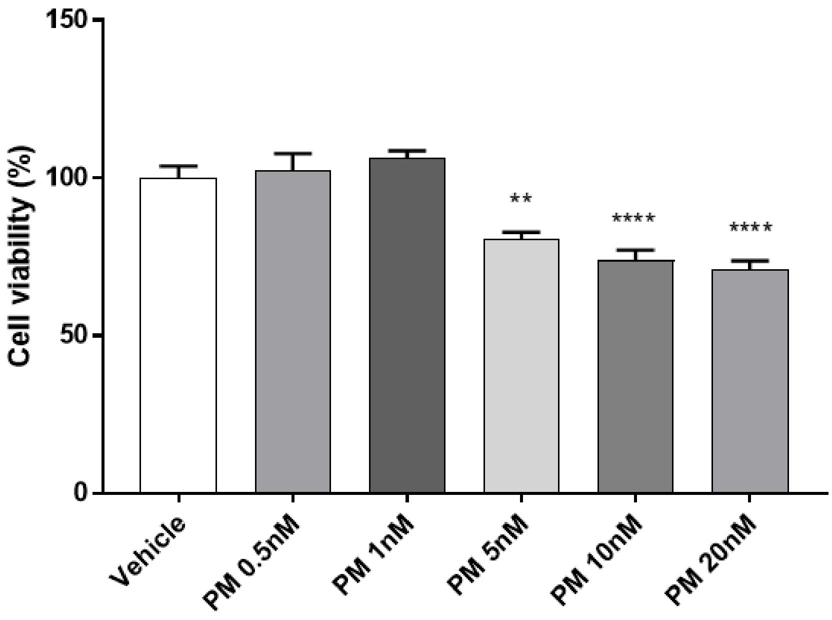

**Figure S2. Effect of different doses of PM01183 on viability of fully differentiated myotubes, after 24-hour treatment**. Cell viability was measured with SRB assays and expressed as percentage of controls, represented by corresponding vehicle-treated cells. Results are plotted as mean ± SEM. n = 8. ** p≤0.01 and **** p≤0.001, one-way ANOVA with post-hoc Dunnett's multiple comparison test.

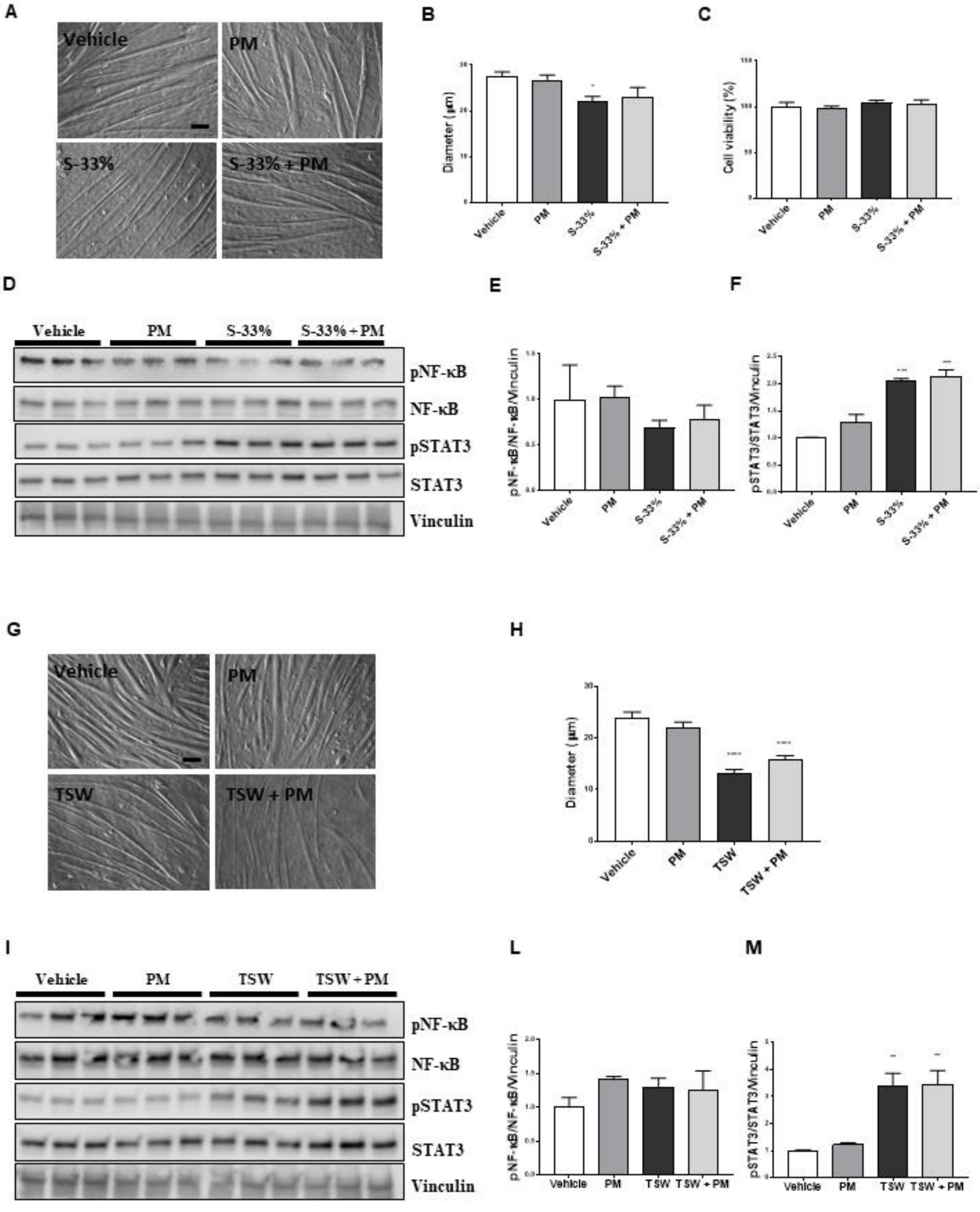


**Figure S3. PM01183 does not directly prevent C26-induced STAT3 activation in cachectic myotubes.** Representative images of myotubes treated with vehicle or 1 nM PM01183 or 33% C26 conditioned medium (S-33%) or in combination (S-33% + PM) for 24 h are shown (scale bar: 100 µm) (A). Myotube diameters were measured manually with ImageJ software, n = 22-43 (B). Cell viability was measured with SRB assays and expressed as percentage of controls, represented by corresponding vehicle-treated cells, n = 6 (C). Western Blot analysis for pNF-κB/NF-κB, pSTAT3/STAT3 is shown (D) and related band quantitation is plotted, n = 3 (E and F). Vinculin was used as loading control. Representative images of myotubes treated with vehicle or 1 nM PM01183 or

with medium conditioned by C26 cells cultured in the Transwell system (TSW) or in combination (TSW + PM) for 24 h are shown (scale bar: 100 µm) (G). Myotube diameters were measured manually with ImageJ software, n = 41-79 (H). Western Blot analysis for pNF-κB/NF-κB, pSTAT3/STAT3 is shown (I) and related band quantitation is plotted, n = 3 (L and M). Vinculin was used as loading control. Results are plotted as mean ± SEM. * p≤0.05, ** p≤0.01, *** p≤0.001 and **** p≤0.0001, one-way ANOVA with post-hoc Tukey's multiple comparison test.

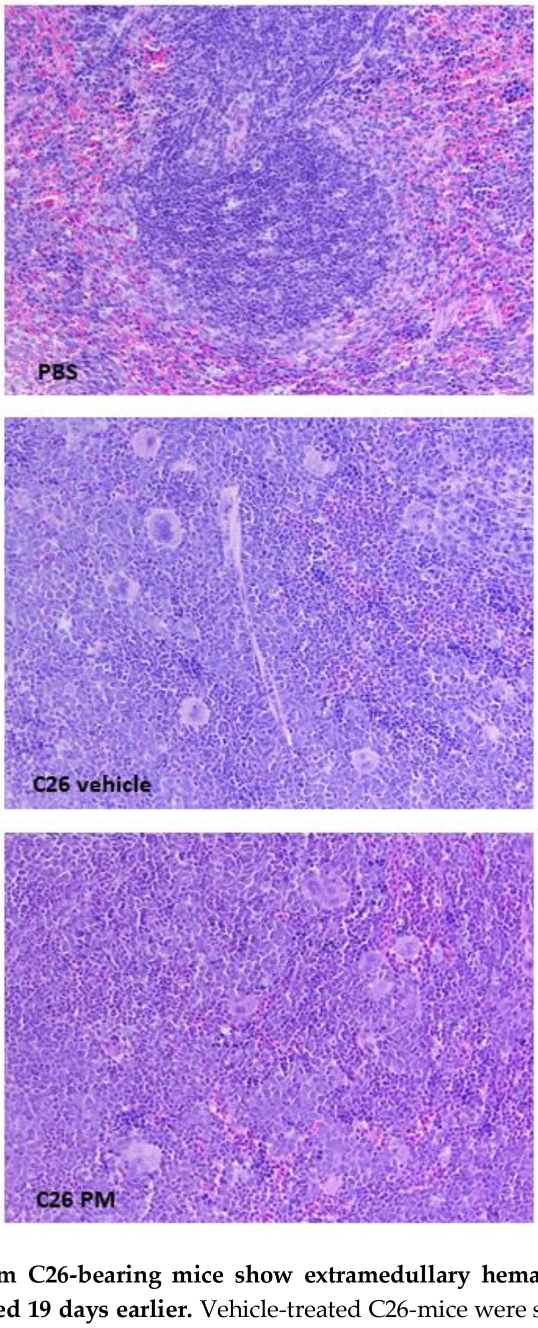


**Figure S4. Spleens from C26-bearing mice show extramedullary hematopoiesis regardless the treatment that was ended 19 days earlier.** Vehicle-treated C26-mice were sacrificed between 24 and 31 days from tumor implant and PM01183-treated C26-mice were euthanized between 34 and 41 days from tumor implant (where the last drug injection was given between 13 and 20 days earlier), n = 3. On the upper panel, it is shown the histological appearance of an example of normal spleen from a PBS-injected mouse: part of follicular structure surrounded by red pulp is shown by Hematoxylin and Eosin's staining. Original magnification: 200x. On the central and lower panels, the histological appearance of a representative spleen from a C26-bearing mouse sacrificed 28 days after tumor implant and that of a PM01183-treated C26-mouse euthanized 40 days after tumor implant are shown. The treatment ended 19 days earlier. In both cases, severe extramedullary hematopoiesis

characterized by the presence of myeloid cells at different degree of differentiation appears with Hematoxylin and Eosin's staining. Original magnification: 200x.

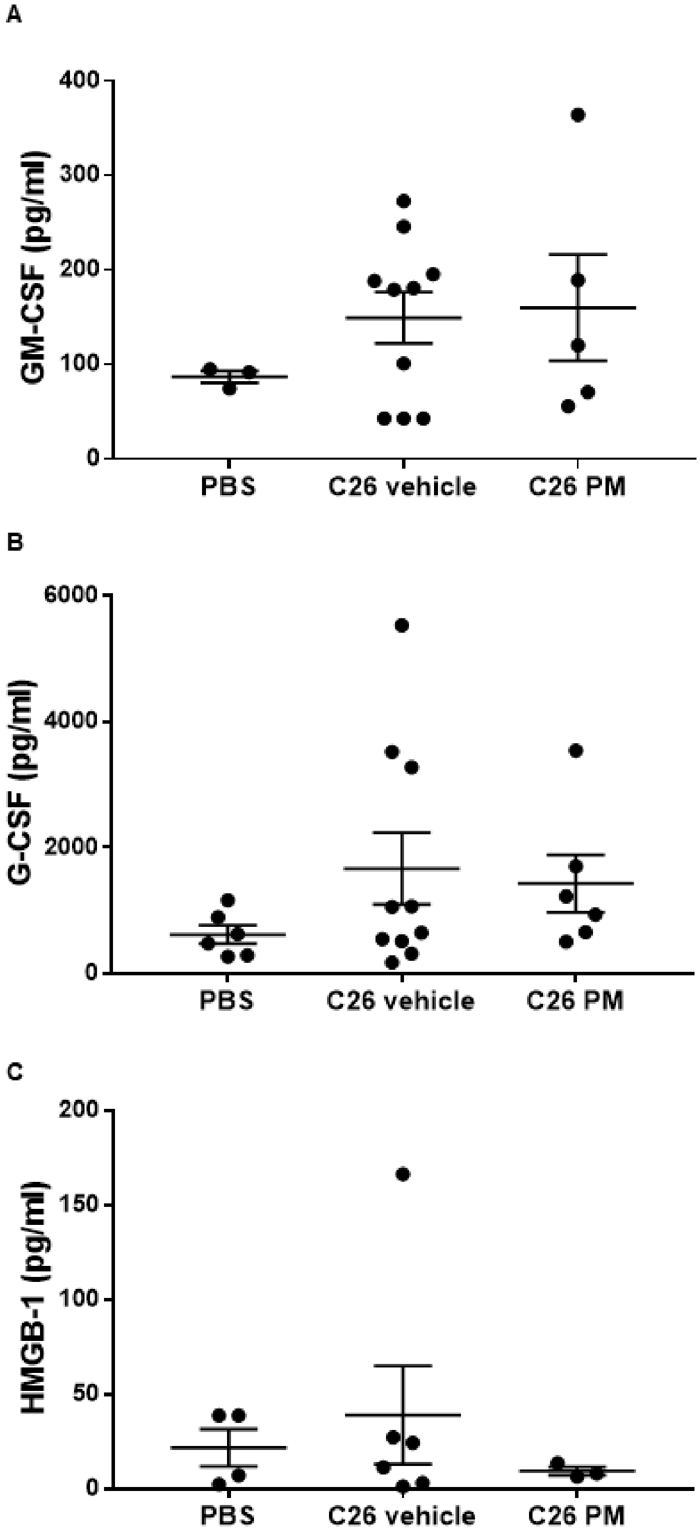


**Figure S5. The circulating levels of GM-CSF, G-CSF and HMGB-1 do not change in C26-mice treated with vehicle or PM01183**. Plasma levels of GM-CSF (A) and G-CSF (B) were measured by multiplex assays, while circulating levels of HMGB-1 were assessed by ELISA (C) in PBS-mice (6/group), C26-mice treated with vehicle (10/group) or PM01183 (6/group) for 10-13 days after tumor injection. PBS-treated mice were used as controls. Results are plotted as mean ± SEM. One-way

ANOVA with post-hoc Tukey's multiple comparison test (A) or Kruskal-Wallis with post hoc Dunn's multiple comparison test (B and C) were performed.

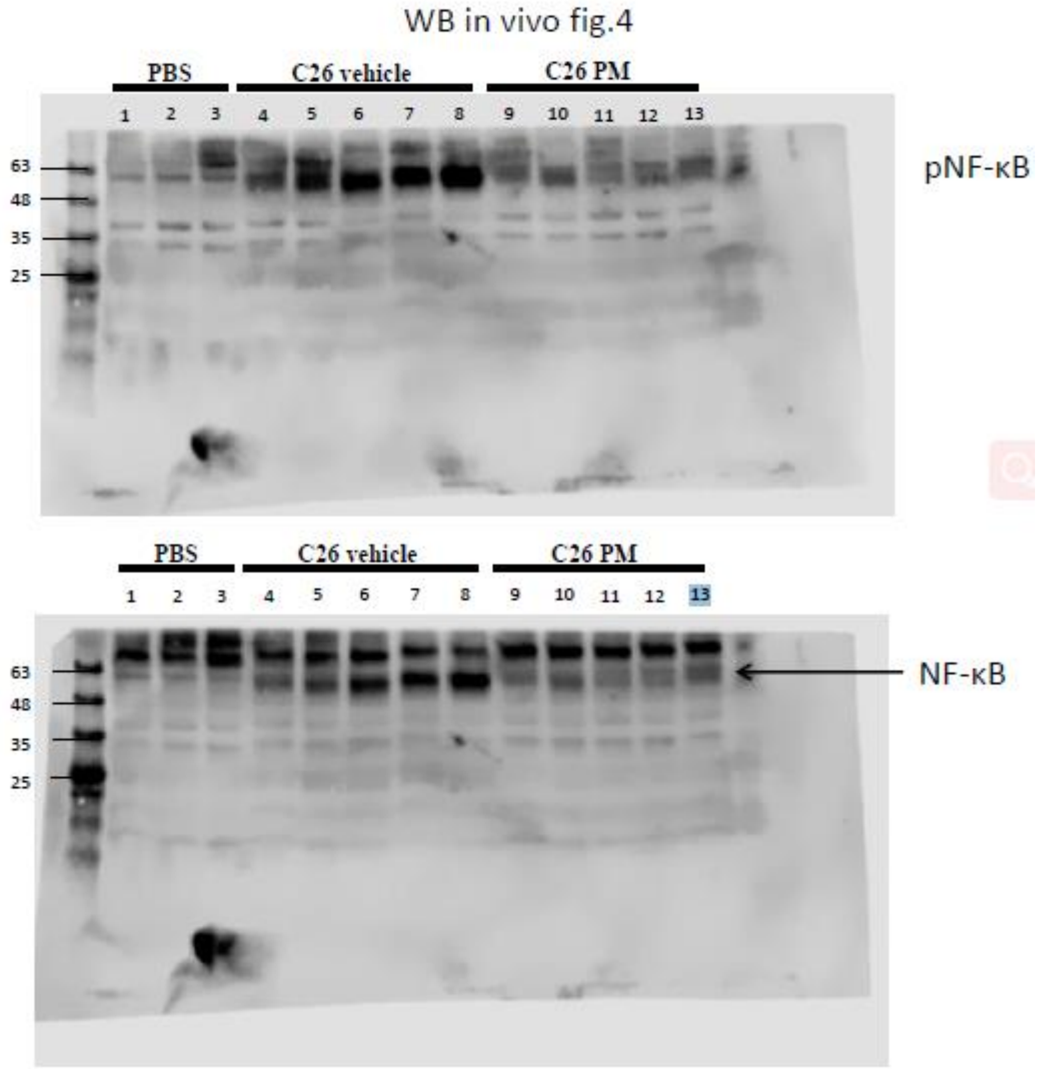

WB in vivo fig.4

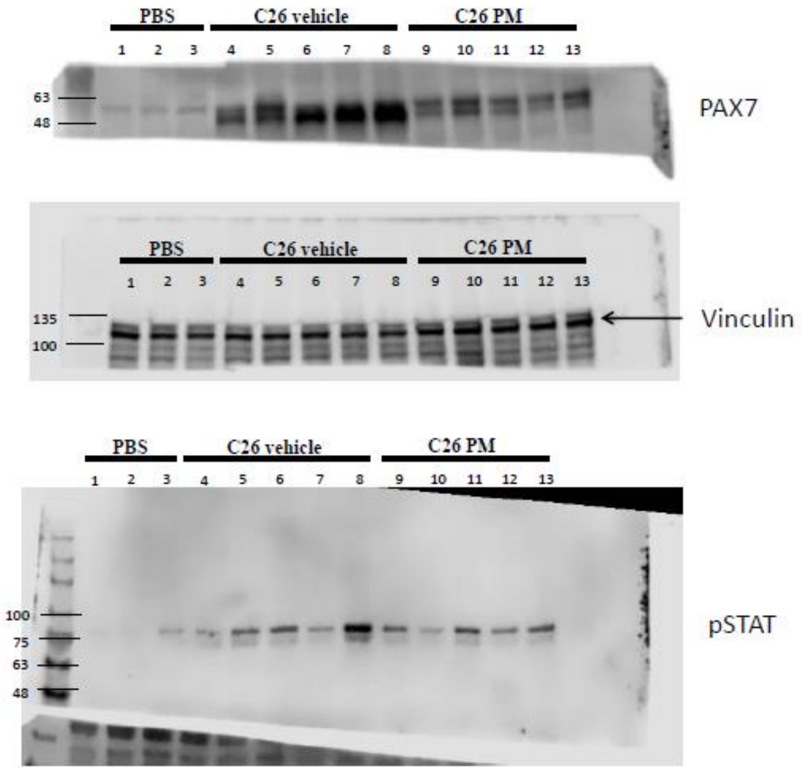


WB in vivo fig.4

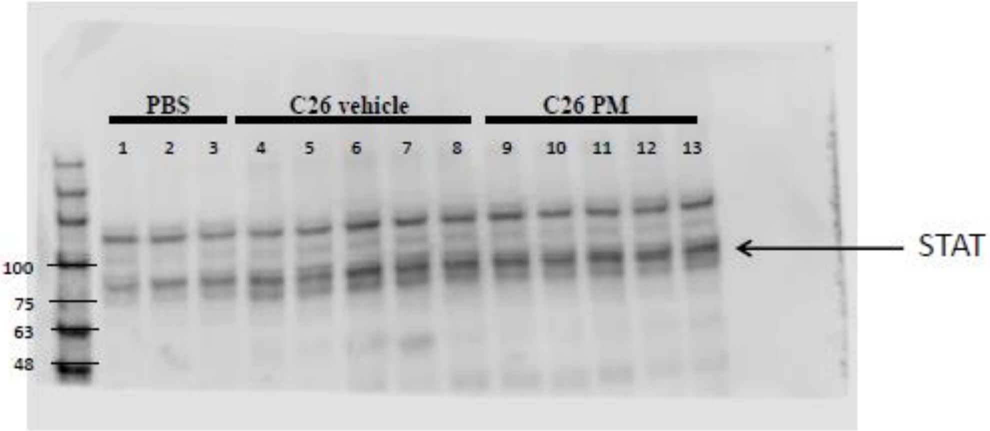

WB in vitro fig.5

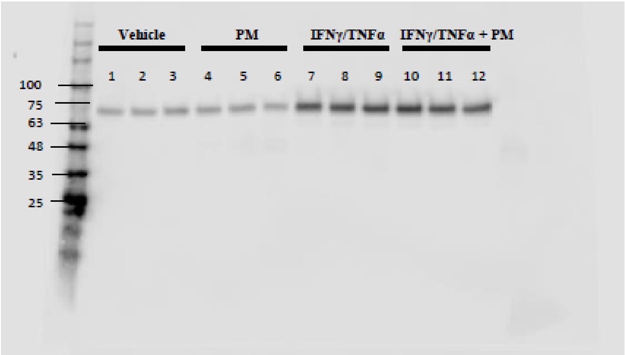


pNF-κB

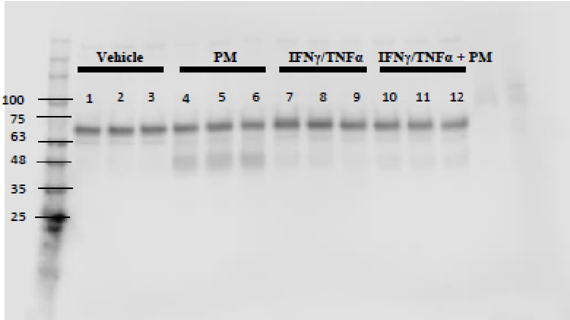


NF-κB

WB in vitro fig.5

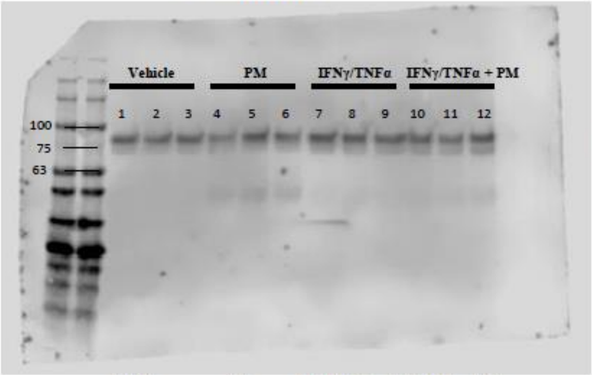


pSTAT

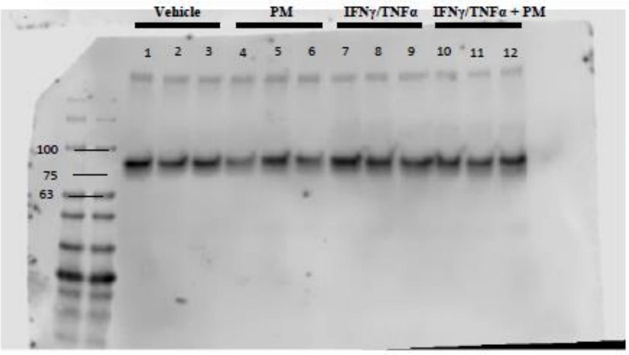


STAT

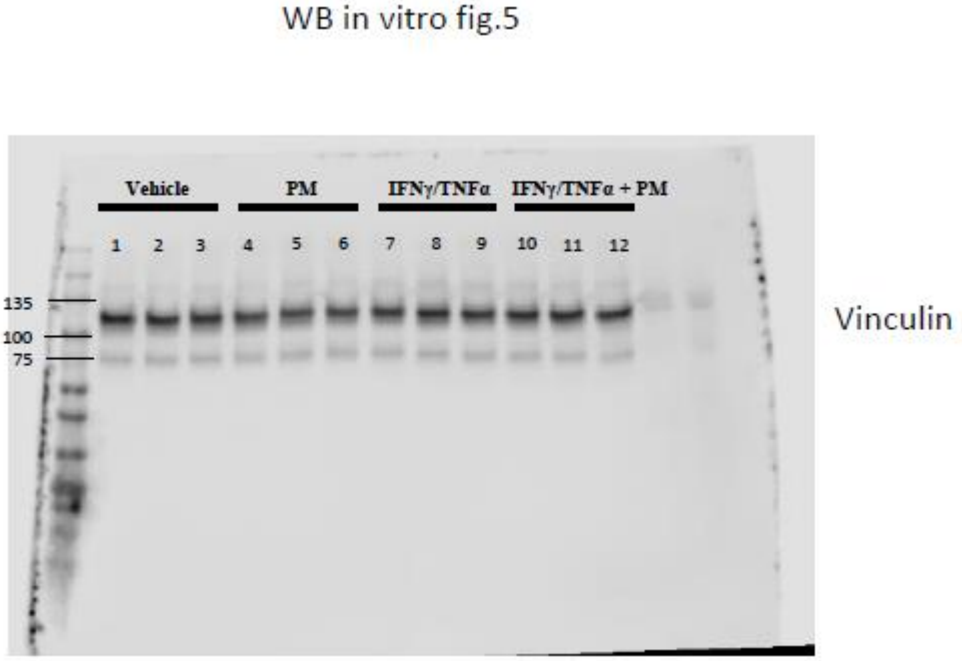


**Figure S6.** The whole western blot image of Figures 4,5.

## Supplementary Methods

*Hematoxylin and Eosin staining*

Ten-µm thick cryosections of gastrocnemius were used for Hematoxylin and Eosin staining. Slides were incubated with Hematoxylin solution for 10 min to stain the nuclei. After a wash with running water, slides were incubated with Eosin solution for 3 min. Finally, slides are washed with 70% ethanol for 20 sec, 90% ethanol for 20 sec, 100% ethanol for 1 min and xylene for 3 min and then, when dried, mounted with a xylene-based mounting medium. Pictures were acquired with a Virtual Slide Microscope VS120 (40X magnification for gastrocnemius, Olympus, Shinjuku, Japan). Similar staining was performed for spleens.

*Fiber Size Measurement*

Ten-µm thick cryosections of gastrocnemius were used for cross sectional area (CSA) measurements in blind conditions by at least two operators using ImageJ software (National Institutes of Health, Bethesda, MA, USA). Pictures of muscle fibers were acquired with an Olympus Microscope IX71 (20× magnification, 10× ocular lens, Olympus, Shinjuku, Japan) with Cell F (2.6 Build1210, Olympus, Shinjuku, Japan) imaging software for Life Science microscopy (Olympus Soft Imaging solution GmbH, Munster, Germany).

*Cell Culture*

On the fourth day of differentiation, myotubes were treated for 24 h with different doses of PM01183 (from 0.5 to 20 nM) and toxicity levels were evaluated by sulforhodamine B (SRB) assay (Sigma, St. Louis, MO, USA), using DMSO as vehicle. To further investigate the effect of PM01183 *in vitro*, myotubes were treated on the fourth day of differentiation for 24h with 1 nM PM01183 or 33% C26 conditioned medium or in combination. C26 cells were seeded at 17000 cells/cm$^2$ 72 h before supernatant collection and grown in differentiation medium for the last 24 h. To characterize the effect of C26 cells on myotubes also in presence of PM01183, we used a Transwell (Corning, NY, USA) co-culture system (as in [1]). C26 cells were seeded at 17000 cells/cm$^2$ in the Transwell insert 72 h before transfer on fully differentiated myotubes and grown in differentiation medium for the last 48 h. Myotubes with C26 cell insert were treated for 24 h with vehicle or 1 nM PM01183.

*Immunoblotting*

The antibodies used are as follows: 1:10000 anti-vinculin (V9264, Sigma, St. Louis, MO, USA), 1:1000 anti-phosphoSer536-NF-κB p65 (3033, Cell Signaling, Danvers, MA, USA), 1:1000 anti-NF-κB p65 (8242, Cell Signaling, Danvers, MA, USA), 1:1000 anti-phosphoTyr705-STAT3 (9145, Cell Signaling, Danvers, MA, USA), 1:1000 anti-STAT3 (9139, Cell Signaling, Danvers, MA, USA).

*Cytokine Measurements*

G-CSF and GM-CSF levels in plasma were measured with MILLIPLEX MCYTOMAG-70K-1 from Merck Millipore (Burlington, MA, USA). The detection range is 3.2-10000 pg/mL. Values below the detection limit were not included in the analyses and related graphs. HMGB-1 levels in murine plasma were measured in an enzyme-linked immunosorbent assay (ELISA), according to manufacturer's protocol (EM0382, FineTest, Wuhan, Hubei, China). Values below the detection limit were not included in the analyses and related graphs. The detection range is 15.625-1000 pg/mL.

## References

1. Jackman, R.W.; Floro, J.; Yoshimine, R.; Zitin, B.; Eiampikul, M.; El-Jack, K.; Seto, D.N.; Kandarian, S.C. Continuous Release of Tumor-Derived Factors Improves the Modeling of Cachexia in Muscle Cell Culture. *Front Physiol* **2017**, *8*, 738, doi:10.3389/fphys.2017.00738.
